# Novel Exenatide Analogs with Peptidic Albumin Binding Domains: Potent Anti-Diabetic Agents with Extended Duration of Action

**DOI:** 10.1371/journal.pone.0087704

**Published:** 2014-02-04

**Authors:** Odile E. Levy, Carolyn M. Jodka, Shijun Steven Ren, Lala Mamedova, Abhinandini Sharma, Manoj Samant, Lawrence J. D’Souza, Christopher J. Soares, Diane R. Yuskin, Li Jenny Jin, David G. Parkes, Krystyna Tatarkiewicz, Soumitra S. Ghosh

**Affiliations:** Amylin Pharmaceuticals LLC, San Diego, California, United States of America; University of Ulster, United Kingdom

## Abstract

The design, synthesis and pharmacology of novel long-acting exenatide analogs for the treatment of metabolic diseases are described. These molecules display enhanced pharmacokinetic profile and potent glucoregulatory and weight lowering actions compared to native exenatide. [Leu^14^]exenatide-ABD is an 88 residue peptide amide incorporating an Albumin Binding Domain (ABD) scaffold. [Leu^14^]exenatide-ABP is a 53 residue peptide incorporating a short Albumin Binding Peptide (ABP). [Leu^14^]exenatide-ABD and [Leu^14^]exenatide-ABP exhibited nanomolar functional GLP-1 receptor potency and were metabolically stable *in vitro* in human plasma and in a pancreatic digestive enzyme mixture. Both molecules displayed picomolar and nanomolar binding association with albumin across multiple species and circulating half lives of 16 and 11 hours, respectively, post a single IV dose in rats. Unlike exenatide, both molecules elicited robust glucose lowering when injected 1 day prior to an oral glucose tolerance test, indicative of their extended duration of action. [Leu^14^]exenatide-ABD was compared to exenatide in a *Lep ^ob/ob^* mouse model of diabetes. Twice-weekly subcutaneously dosed [Leu^14^]exenatide-ABD displayed superior glucose lowering and weight loss in diabetic mice when compared to continuously infused exenatide at the same total weekly dose. A single oral administration of each molecule *via* an enteric coated capsule to cynomolgus monkeys showed superior pharmacokinetics for [Leu^14^]exenatide-ABD as compared to [Leu^14^]exenatide-ABP with detectable exposure longer than 14 days. These studies support the potential use of these novel long acting exenatide analogs with different routes of administration for the treatment of type 2 diabetes.

## Introduction

Peptide hormone-based therapeutics deliver significant therapeutic benefits while displaying exquisite receptor selectivity. While insulins have long been a treatment mainstay for diabetes, the development of peptide drugs from somatostatin [1], calcitonin [2], [3], parathyroid hormone (PTH) [4], vasopressin [5], and glucagon like peptide-2 (GLP-2) [6] classes have become important treatment options for metabolic indications. With the recent introduction of Byetta™, Victoza™, Lyxumia® and Bydureon™, the GLP-1-based drug class has emerged as an important therapeutic regimen for treating patients with type 2 diabetes. These anti-diabetic agents illustrate product differentiation based on differences in efficacy, tolerability, frequency of administration and device presentation.

Long-acting injectable peptide drugs, given by injection, afford greater convenience and promote increased compliance due to less frequent administration. The design of such drugs needs to address proteolytic degradation and renal filtration which plays a significant role in their clearance from the bloodstream. Efforts to reduce renal clearance have focused on increasing molecular size beyond the 60 kDa renal threshold [7] through the covalent fusion of the bioactive molecule to molecular scaffolds such as polyethylene glycol (PEG) polymers [8], [9], [10], [11], XTEN [12], [13], polysaccharides, large natural proteins such as Fc [14], [15], [16] and albumin [17].

Alternate strategies involve modification of peptide and protein drugs to allow a reversible non-covalent association to serum albumin, resulting in half life extension commensurate with their albumin binding affinity [18], [19], [20], [21]. These approaches take advantage of the high abundance of serum albumin in mammalian sera, its wide tissue distribution and extended half-life resulting from its large molecular size and neonatal Fc receptor (FcRn) mediated cell recycling [22], [23], [24]. The half-life of serum albumin is directly proportional to size of species, as exemplified by half-lives in rabbits and humans of 4.6–6.2 and 19 days, respectively [25]. Thus, fatty acid acylation of peptides has emerged as a successful half-life extension approach *via* non-covalent association of the fatty acid alkyl moiety with albumin, and is exemplified by the once-daily anti-diabetic drugs Insulin detemir [26], [27] and liraglutide [28], [29]. Their protracted effect is attributed to drug self association that delays absorption, and their intermittent association with circulating albumin that also protects against enzymatic degradation.

Our half-life extension strategy focused on the fusion of bio-active peptides with small peptidic motifs that display high affinity for albumins across species. Albumin Binding Peptides (ABP) and Albumin Binding Domains (ABD) have been reported in the literature. Dennis *et al* [19] have used phage display to identify a short albumin-binding peptide, that when recombinantly fused to an immunoglobulin fragment D3H44-Fab, improved its half life by 37 fold in rabbits and 26 fold in mice. In addition, an array of bacterial proteins showing high, and specific, affinity for various human plasma proteins have been identified and structural studies have elucidated homologous domains involved in the specific interaction with human albumin [30], [31]. Linhult, Johansson and Jonsson have reported on an ABD from the bacterial protein Streptococcal G (strain 148) that can be molecularly tuned to bind with nanomolar to femtomolar affinity to albumin [32], [33], [34]. The binding of ABD to albumin was shown not to interfere with albumin’s pH-dependent interaction with FcRn that is critical for its recycling mechanism [35].

In our studies, we utilized a previously reported ABP and an ABD licensed from Affibody AB (ABD035) as fusion partners for [Leu^14^]exenatide to generate potent, metabolically stable and long acting exenatide analogs with prolonged glucoregulatory action. The ABP and the ABD components are 1.4 and 5 kDa’s, respectively, and confer extended circulation half-lives to the conjugates through tight, reversible binding to plasma albumin. Our fusion strategy is amenable to chemical or recombinant manufacturing processes, and results in significantly smaller conjugates compared to those with molecular scaffolds such as Fc, XTEN and PEG. They display comparable half-life extension with preservation of biological potency, afford the ability to screen the molecules in animal models from different species and offer flexibility to deliver the fusion molecules by alternate routes of administration.

As a proof of concept study, an oral formulation of [Leu^14^]exenatide-ABD and [Leu^14^]exenatide ABP was administered to cynomolgus monkeys. The preliminary pharmacokinetics results highlight the potential to provide novel exenatide analogs for daily and potentially weekly oral administration.

## Materials and Methods

### Ethics Statement

All studies were approved by the Institutional Animal Care and Use Committee at Amylin Pharmaceuticals, LLC, and at Xenometrics LLC, in accordance with the Animal Welfare Act, AAALAC, and PHS regulations and/or guidelines.

### Reagents

Liraglutide (Victoza® pen) was obtained from Cedar Pharmacy (Henderson, NV, www.cedarpharmacy.com) and dissolved in normal saline for use in mouse studies, or in assay buffer for the GLP-1 receptor functional assay. Synthetic human GLP-1 (7–36) and exenatide were purchased from Bachem (Torrance, CA, USA).

### Animals

All animals were acclimated to the testing facility for at least 1 week prior to dosing. Rodent studies were conducted at Amylin Pharmaceuticals (San Diego, CA, USA) using female 14–20 week old Hsd:NIHS mice (n = 9–16 per group, body weight 31.1±0.2 g) (Harlan, Indianapolis, IN, USA), male 8-week old B6.V-Lep^ob^/J (*ob/ob*) mice (n = 10 per group, body weight 43.9±0.3 g) (Jackson Laboratories Bar Harbor, ME), or male 13–14 week old Sprague Dawley (Hsd) rats (n = 4 per group, body weight 361.9±2.8 g) (Harlan, Indianapolis, IN). Rodents were housed 1–3 per cage at 21–24°C with a 12 h light/dark cycle with ad libitum access to food and water with the exception of tests where fasting (no more than 16 h) was required. Non-human primate studies were out-sourced to Xenometrics (Stilwell, KS, USA). Adult, male non-naïve cynomolgus monkeys (Macaca fascicularis) were obtained from SNBL USA or Covance (6.63+/−0.50 years of age; 6.0+/−1.1 kg, n = 3 per group), and were singly housed in stainless steel caging with slatted flooring, in a conventional facility room with other Cynomolgus monkeys. Animal rooms were environmentally controlled (temperature, humidity and lighting) with continuous access to drinking quality water and a set amount of certified monkey biscuits. Enrichment was provided per SOP, 3–4 days per week and included stimulation of all the senses on a rotating basis. Welfare-related assessments were made on a daily basis by animal care and use technicians and an on-site veterinarian is notified of any health, welfare or behavioral issues. No intervention was necessary for the study. All housing, feeding and environmental enrichment procedures were conducted in compliance with the Animal Welfare Act, AAALAC, and PHS regulations and/or guidelines and Xenometrics SOP’s.

### Peptide Synthesis

[Leu^14^]exenatide (also described as AC3174 in prior reports [36]), is an exenatide analog with leucine substituted for methionine at position 14 to prevent oxidation, and is very similar in its biological activity when compared to exenatide. The design strategy for our peptides was the C-terminal extension of [Leu^14^]exenatide with either the 46 amino acid sequence of ABD035 or the 11 amino acid sequence of ABP (Table 1) *via* a tri-peptide linker. Both exenatide and [Leu^14^]exenatide are interchangeably referred to as parent peptides in this manuscript.

**Table 1:** Names and Sequences of Compounds.

Compound	Sequence
GLP-1 (7–36)	HAEGTFTSDVSSYLEGQAAKEFIAWLVKGR-NH_2_
Exenatide	HGEGTFTSDLSKQMEEEAVRLFIEWLKNGGPSSGAPPPS-NH_2_
[Leu^14^]exenatide	HGEGTFTSDLSKQLEEEAVRLFIEWLKNGGPSSGAPPPS-NH_2_
[Leu^14^]exenatide-ABP	HGEGTFTSDLSKQLEEEAVRLFIEWLKNGGPSSGAPPPSGGGDI-cyclo[CLPRWGC]LW-OH
[Leu^14^]exenatide-ABD	HGEGTFTSDLSKQLEEEAVRLFIEWLKNGGPSSGAPPPSGGSLAEAKVLANRELDKYGVSDFYKRLINKAKTVEGVEALKLHILAALP-OH
Liraglutide	HAEGTFTSDVSSYLEGQAA[N-epsilon(gamma-Glu(N-alpha-hexadecanoyl))-Lys]EFIAWLVRGRG-OH
ABD035	LAEAKVLANRELDKYGVSDFYKRLINKAKTVEGVEALKLHILAALP-OH

[Leu^14^]exenatide-ABD was assembled on Fmoc-Pro-Novasyn TGT resin (EMD Chemicals, Gibbstown, NJ) using standard solid phase peptide synthetic protocols on a Prelude automated peptide synthesizer (Protein Technology Inc, Tucson, AZ, (0.2 mmole scale)). Fmoc amino acid residues were activated using 4.0 eq of 0.5 M O-(7-azabenzotriazol-1-yl)-N,N,N′,N′-tetramethyluronium hexafluorophosphate, 8.0 eq of 1 M diisopropylethylamine in dimethylformamide (DMF) and double coupled for 1 hour. Fmoc group was removed by treatment with 20%(v/v) piperidine in DMF. Cleavage and deprotection was performed by treatment of the resin with reagent B (93% trifluoroacetic acid (TFA), 3% phenol, 3% water and 1% triisopropylsilane) for 3 hours. The cleaved peptide was precipitated using t-butyl methyl ether, pelleted by centrifugation and lyophilized. The pellet was re-dissolved in 10% acetonitrile/water (10–15 min), filtered and purified *via* reverse phase HPLC using C5 column and an acetonitrile/water gradient containing 0.1% TFA. The compound was re-purified *via* reverse phase HPLC C18 column using a shallower gradient. The purified compound was analyzed by ESI-MS (empirical weight 9462.86; mass found 9461.39). The pure peptide obtained as a TFA salt was used for all biological evaluations.

Similarly, [Leu^14^]exenatide-ABP was assembled on Fmoc-Trp(Boc)-Novasyn TGT resin using standard solid-phase peptide synthetic protocols on a Prelude ™ peptide synthesizer on a 0.2 mmole scale. After cleavage of the peptide from the resin, the lyophilized material was re-dissolved in 20% acetic acid (7–10 mg/ml) and oxidized with an iodine solution (0.125 M in acetic acid) for 10–15 minutes, filtered and purified *via* reverse-phase HPLC using C5 column and an acetonitrile/water gradient containing 0.1% TFA. The purified product was analyzed by ESI-MS and re-purified via reverse phase HPLC C18 column using a shallower gradient. Final peptide was analyzed by ESI-MS (empirical weight 5682.4; mass found 5681.76). The pure peptides thus obtained as a TFA salt were used for all biological evaluations.

### Albumin Binding Affinity

Characterization of the binding of our molecules to albumin was conducted by Biosensor Tools, LLC (Salt Lake City, UT) with a BioRad ProteOn XPR36 system 10 (Bio-Rad Laboratories, Hercules CA, USA), using a GLC sensor chip at 25°C. Using amine coupling, following the manufacturer’s instructions, human, rat, mouse, dog and monkey albumins were captured between 2000 and 5000 resonance units (RU).

Binding of the engineered peptides was tested as follows using 3 concentrations in duplicate: 100 µM, 33.3 µM and 11.1 µM for [Leu^14^]exenatide-ABD and 3.1 µM, 1.03 µM and 0.344 µM for [Leu^14^]exenatide-ABP. The highest concentration was allowed to disassociate for 3 hours. The running buffer contained 10 mM HEPES pH 7.4, 150 mM NaCl, 3 mM EDTA, 0.005% Tween-20 and 0.2 mg/ml ovalbumin. A 12-second pulse of 1/200 dilution of phosphoric acid was used to completely regenerate the surfaces between samples. After subtraction of reference surface and buffer injection, curves were fitted to a 1∶1 (Langmuir) binding model using BIA evaluation software (BioSensor Tools) with correction for mass transfer and with RU max set as a local parameter.

### GLP-1 Receptor Functional Assay

Peptides were diluted in assay buffer (HBSS, 0.1%BSA) and incubated with 6–23 clone 6 cells endogenously expressing the rat GLP-1 receptor, in the presence of 250 µM IBMX (Calbiochem 410957). Following 30 minutes incubation, the cAMP was measured using the cAMP Dynamic 2 assay (Cisbio US, Bedford, MA) as per the manufacturer’s instructions. cAMP was detected by a decrease in time-resolved fluorescence energy transfer using a GeniousPro plate reader (Tecan US, San Jose, CA). To test whether [Leu^14^]exenatide-ABD’s *in-vitro* activity was affected by albumins, we also performed this assay in the presence of 1% serum albumin of different species (bovine, human and rat).

### Stability in Human Plasma

Standard solutions of the peptides (200 µM in PBS) were prepared and diluted in human plasma (1 ml; Bioreclamation, Long Island, NY) to a final concentration of 50 µM at 37°C. At 0, 1, 2, 3, 4, and 5 h, aliquots of 20 µl were removed, quenched with 5% phosphoric acid (100 µl ) and the samples were analyzed as triplicates by using analytical HPLC coupled with a mass detector. [Leu^14^]exenatide and GLP-1 were used as positive and negative controls. The final results were normalized to the positive control and expressed as percent peptide remaining.

### Stability in Pancreatic Digestive Enzyme Mix

Stability of peptides was also assessed in a mixture of amylase, lipase and protease (pancreatin, Cat.# P7545-25 g, Sigma-Aldrich, St. Louis, MO), which are digestive enzymes produced by pancreatic exocrine cells. Peptides (30 mM) in 50 mM phosphate buffer (pH 6.8) and citrate buffers (pH 4 & pH 5) were incubated for 2 hours in pancreatic mixture at 37°C (20∶1 ratio of peptide to pancreatic mixture) (n = 3). The enzymatic reaction was stopped with the addition of 0.2 N HCl (final concentration of peptide 10 mM) at each time point. Samples taken at 0, 10, 20, 30, 60, and 120 min were analyzed for peptide stability by reverse-phase HPLC.

### Intravenous (IV) Pharmacokinetics in Rats and Non-Human Primates

In all studies, blood samples were collected into tubes containing EDTA and were kept at approximately 5°C until and during centrifugation. Plasma, collected from the centrifuged blood samples, was stored at −70°C until analyzed for peptide concentration. Male drug-naïve Sprague Dawley rats received 2 nmol/kg of exenatide or [Leu^14^]exenatide-ABD or [Leu^14^]exenatide-ABP in phosphate buffer (PBS) as a bolus into the tail vein.

Monkeys were fasted overnight prior to dosing, and through approximately 2 hours post-dose, and received [Leu^14^]exenatide-ABD, 2.4 nmol/kg in pH 4 acetate buffer or [Leu^14^]exenatide-ABP, 2.4 nmol/kg in pH 7.4 phosphate buffer administered through an indwelling catheter in the cephalic vein. The test articles were supplied in ready to use IV solutions. All animals were dosed in the morning (09∶09–09∶33) and blood samples collected while in a restraint chair. The animals were housed in their standard cages between sample collections. Blood samples were collected from the cephalic or saphenous veins pre-dose and at various time points up to 14 days post-dose. The study consisted of 4 experimental groups containing 3 male nonhuman primates each dosed in a parallel design. The animals were selected based on their availability with no randomization being performed. The animals used came from the Xenometrics non-naïve colony. No animals were sacrificed in the course of this study and all animals were returned to the non–naïve stock colony following completion of the study.

### Oral Glucose Tolerance Test (OGTT) in Mice

All peptides were injected IP at t = −5 min into 4-hour fasted Hsd:NIHS female mice. Glucose gavage (1.5 g/kg) was given at t = 0. Blood samples were taken from the tail at t = 30 min. In addition, peptides were administered 1 day prior to the glucose challenge to evaluate their duration of action in an OGTT.

### Sub-chronic Dosing in Diabetic *Lep^ob/ob^* Mice

Mice were singly housed and randomized into three treatment groups based on glycated hemoglobin A1c (HbA1c) prior to the 4-week study. [Leu^14^]exenatide-ABD at 100 nmol/kg/dose in PBS was administered IP twice a week (BIW). To match the [Leu^14^]exenatide-ABD total weekly dose, exenatide was administered at 30 nmol/kg/d in 30 mM Na-acetate buffer (pH 4.5) *via* continuous SC infusion using osmotic mini-pumps (ALZET® model 2002, Durect Corp., Cupertino, CA). To assure the same animal handling across all groups, [Leu^14^]exenatide-ABD-treated mice received mini-pumps with infusion vehicle, the exenatide-treated group was injected BIW with PBS, and vehicle control mice were dosed BIW with PBS and implanted with mini-pumps delivering infusion vehicle. After 2 weeks, all mice were re-implanted with new mini-pumps with the same treatment administered for the next 2 week. HbA1c and body weight were assessed periodically. Following the study, all mice were sacrificed with an overdose of isoflurane.

### Formation of Enteric Coated Capsule for Oral Delivery in Non-Human Primates

Sodium chenodeoxycholate in water (0.12 M) was sealed in a glass container and heated. After dissolution was complete, propyl gallate (0.12 M) and sucrose (0.06 M) were added. The solution was allowed to cool to room temperature and pH was adjusted to 7.4 using 1 N NaOH solution. Test peptide and water were added to obtain a final concentration of the test peptide (0.02 mM). The solution was lyophilized and the dry powder was filled into gelatin capsule (size 3, Torpac, Fairfield, NJ). The filled capsules were subsequently enteric coated with a suspension consisting of Eudragit L100-55, triethyl citrate, and talc in a mixture of isopropyl alcohol, acetone and water according to guidelines provided by the capsule manufacturer.

### Pharmacokinetic Studies in Non-Human Primates

Cynomolgus monkeys were dosed PO with either [Leu^14^]exenatide-ABP or [Leu^14^]exenatide-ABD with a single enteric coated capsule (equivalent to 41 nmol/kg exenatide), followed by up to a 5 ml flush of water to ensure delivery completion. Blood samples were collected from the cephalic or saphenous veins predose and at 0.25, 0.5, 1, 2, 3, 4, 8, 24 and 48 h post-dose and on days 5, 7, 10, 12, and 14. A half banana was given following the 2 h PK sample collection, ½ daily ration was given ∼4 h post-dose and the remaining daily rations were fed ∼6 h post-dose. Absolute bioavailability was calculated by dividing the dose-adjusted AUC of the PO PK curve by the AUC of the IV PK curve.

### Biochemical Analysis

Plasma glucose was measured using a OneTouch^®^ Ultra^®^ blood glucose meter (LifeScan, Inc., Johnson & Johnson Co, Milpitas, CA). Whole blood HbA1c was measured using the Olympus AU680 clinical analyzer (Olympus America, Irving, TX). Plasma concentrations of peptides were measured using a validated two-site “sandwich” ELISA as described previously [37] using two monoclonal antibodies that target the N- and C- terminal regions of exenatide. The linear range of the standard curve was 40–5,120 pg/ml, with the lower limit of quantitation at 60 pg/ml and the upper limit of quantitation at 5,000 pg/ml.

### Statistical Analyses

Results are presented as means ± SEM, graphed and analyzed using GraphPad Prism 5^®^. The potency of peptides in the *in vitro* functional assay and in an OGTT was determined by the analysis of concentration-response curves using non-linear regression analysis. Statistical differences (p<0.05) between groups in the sub-chronic *Lep^ob/ob^* mouse study were identified with one-way ANOVA followed by post-hoc Tukey’s test. Albumin binding data shown in Table 2 are presented as mean ± SD.

**Table 2:** Binding Constants for Albumin Interactions at 25°C.

Albumin	*k*_a_ (M^−1^s^−1^)	*k*_d_ (s^−1^)	*K*_D_
**ABD035**			
Human	3.4(1)×10^6^	5.4(1)×10^−5^	16.1(4) pM
Dog	5.3(2)×10^6^	0.00106(4)	201(2) pM
Monkey	2.06(4)×10^6^	2.52(5)×10^−4^	123(1) pM
Mouse	4.0(3)×10^6^	0.0049(4)	1.24(1) nM
Rat	9.9(3)×10^5^	1.81(2)×10^−5^	18.3(5) pM
**[Leu^14^] exenatide-ABP**			
Human	1.8(1)×10^4^	0.029(2)	1560 nM
Dog	5.6(3)×10^4^	0.025(1)	450 nM
Monkey	9.3(4)×10^4^	0.056(2)	610 nM
Mouse	1.16(4)×10^5^	0.0178(7)	150 nM
Rat	8.6(3)×10^4^	0.0180(5)	210 nM
**[Leu^14^] exenatide-ABD**			
Human	1.51(3)×10^6^	7.47(6)×10^−6^	4.94(9) pM
Dog	4.65(6)×10^6^	4.56(6)×10^−4^	98.1(4) pM
Monkey	7.5(1)×10^5^	3.03(2)×10^−5^	40.2(2) pM
Mouse	1.61(2)×10^6^	4.07(4)×10^−4^	253.3(6) pM
Rat	2.65(1)×10^5^	2.18(6)×10^−6^	8.2(2) pM

The number in parentheses represents the standard deviation in the last significant digit.

## Results

### Albumin Binding Affinity

Binding constants (overall affinity K_D_ (pM), association rate constant k_a_ (M^−1^s^−1^) (k_on_) and dissociation rate constant k_d_ (s^−1^) (k_off_)) determined for [Leu^14^]exenatide-ABP and [Leu^14^]exenatide-ABD to albumin from different species are presented in Table 2. The result show that both peptides associate with serum albumins of all species with high affinity. In general, the affinity of the ABD fused exenatide molecule to albumins was several orders of magnitude higher than its ABP fused exenatide counterpart. With respect to human albumin an association rate constant k_a_ of 1.51±0.03×10^6^ M^−1^s^−1^ and a dissociation rate constant k_d_ of 7.47±0.06×10^−6 ^s^−1^ (based on a 3 h measurement), resulted in an overall affinity K_D_ of 4.94±0.09 pM for [Leu^14^]exenatide-ABD. This value corresponds closely to that of the unconjugated ABD035 (K_D_ 16.1±0.4 pM).

### *In Vitro* Activity and Stability of Peptides

**Figure 1:**
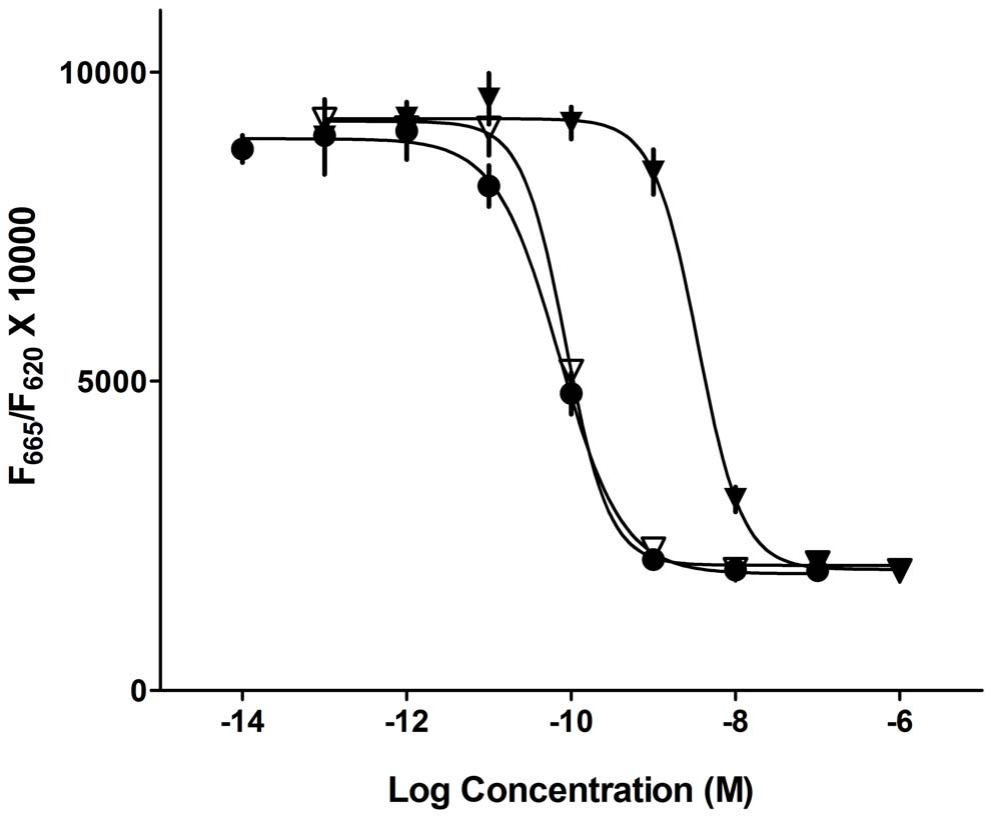
Functional activity at the GLP-1 receptor. GLP-1 (7–36) (▪), [Leu^14^]exenatide-ABD (▾) and [Leu^14^]exenatide-ABP (▿). GLP-1 (7–36) was used as a reference standard in the assay. The assay was run in quadruplicates and data are presented as mean ± SD. Abbreviations: Fluorescence (F).

Results from *in vitro* studies are summarized in Table 3. [Leu^14^]exenatide-ABP and [Leu^14^]exenatide-ABD were functionally active, with an EC_50_ of 0.066 nM and 0.63 nM, respectively in a GLP-1 receptor functional assay that contained 0.1% BSA in the assay medium (Figure 1). [Leu^14^]exenatide-ABD receptor functional activity was not affected by the presence of higher concentrations of serum albumin of different species (EC_50_’s obtained in the presence of 1% serum albumin: bovine 0.23 nM, human 0.21 nM, rat 0.24 nM). The 100-fold reduction of potency in the receptor functional assay for [Leu^14^]exenatide-ABD when compared to [Leu^14^]exenatide (0.006 nM) is possibly due to steric hindrance caused by the ABD fused to the C-terminus of the peptide. Similar to the parent peptides, [Leu^14^]exenatide-ABD was stable in human plasma over 5 hours. However some degradation of [Leu^14^]exenatide-ABP was noted. Significant degradation of all peptides occurred at pH 6.8 when exposed to a mixture of pancreatic enzymes. The stability in the pancreatin mix improved with the decrease of pH and degradation was negligible in the most acidic milieu (pH 4.0) for all peptides.

**Table 3:** *In Vitro* Activity, Metabolic Stability, and Plasma Glucose in OGTT.

Compound	GLP-1 Receptor Functional Assay	5 h Stability in Human Plasma (%)	2 h Stability in Pancreatin Mix (%)			OGTT		OGTT DOA
	EC_50_ (nM)		pH 4.0	pH 5.0	pH 6.8	ED_50_ (nmol/kg)	Maximal Efficacy vs. Vehicle (%)	Maximal Efficacy vs. Vehicle (%)
GLP-1 (7–36)	0.010							
Exenatide	0.004	100±3	95±3	100±4	42±2	0.4	−43	not active
[Leu^14^]exenatide	0.006	100±5	100±3	98±3	42±3	0.9	−33	not active
[Leu^14^]exenatide-ABP	0.066	82±1	98±3	95±2	65±3	8	−38	−19
[Leu^14^]exenatide-ABD	0.63	100±5	100±3	62±2	33±2	3	−29	−22
Liraglutide	0.35							−18
ABD035	not active	no data	no data	no data	10±2	not active	not active	not active

Stability results (measured as plasma AUC 0–5 h; pancreatin mix AUC 0–2 h)) are expressed as a percentage of peptide remaining versus stable control peptide ± SEM. Replicates within each assay n = 3, except for exenatide and [Leu^14^]exenatide-ABP where n = 2.

### Intravenous (IV) Pharmacokinetics in Rats and Non-Human Primates

**Figure 2:**
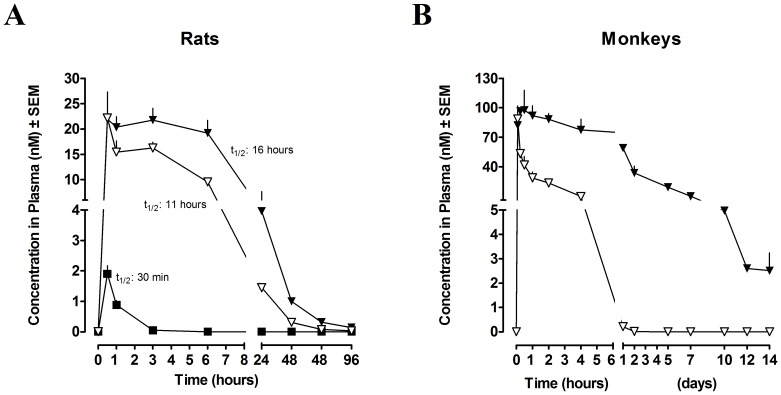
PK profile of intravenously dosed peptides in rats and monkeys. (A) rats, n = 3–4 and (B) monkeys, n = 3. Exenatide (▪), [Leu^14^]exenatide-ABD (▾) and [Leu^14^]exenatide-ABP (▿). Data are presented as mean ± SEM.

The pharmacokinetic (PK) profiles of the compounds intravenously dosed in rats were markedly extended versus the PK profile of exenatide. In rats, the half-life of the un-conjugated exenatide was 30 minutes, whereas the absolute calculated half-lives for [Leu^14^]exenatide-ABP and [Leu^14^]exenatide-ABD were 11 and 16 hours, respectively (Figure 2A). Furthermore, a single IV injection of [Leu^14^]exenatide-ABP or [Leu^14^]exenatide-ABD in monkeys showed detectable exposure up to two days and greater than 14 days, respectively (Figure 2B).

### OGTT in Mice

When dosed 5 min prior to an oral glucose challenge at 25 nmol/kg, [Leu^14^]exenatide-ABP and [Leu^14^]exenatide-ABD blunted the rise in plasma glucose relative to vehicle-treated mice with efficacy comparable to exenatide or [Leu^14^]exenatide (Table 3). However, when dosed 24 h prior to an oral glucose challenge at 25 nmol/kg, only [Leu^14^]exenatide-ABP and [Leu^14^]exenatide-ABD blunted the plasma glucose excursion relative to vehicle, indicative of their long duration of action. Liraglutide at the same dose had a similar glucose lowering effect. Exenatide was ineffective in this assay even at much higher doses (250 nmol/kg).

### Sub-chronic Dosing in Diabetic *Lep^ob/ob^* Mice

**Figure 3:**
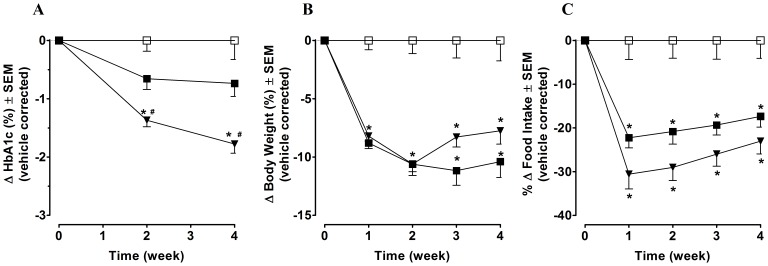
Four-week chronic dosing of peptides in diabetic *Lep^ob/ob^* Mice. (A) effects on HbA1c (B) effects on body weight and (C) effects on food intake. Vehicle (□), [Leu^14^]exenatide-ABD (▾) and exenatide (▪). Data are presented as mean ± SEM, n = 10. *p<0.05 vs. vehicle, #p<0.05 vs. exenatide infusion by ANOVA with Tukey’s test.

*Lep^ob/ob^* mice were diabetic and obese prior to treatment, with a mean HbA1c value of 6.90±0.11% and a mean BW of 43.7±0.6 g. HbA1c values in control vehicle-treated mice increased from baseline by 1.59±0.33% at the end of the 4 week study. Twice-weekly SC injections of [Leu^14^]exenatide-ABD for 4 week resulted in a significant vehicle-corrected reduction in HbA1c (−1.78±0.16%) *vs*. continuous infusion of exenatide at a similar total weekly dose of 210 nmol/kg/week (−0.55±0.27%) or vehicle (p<0.05) (Figure 3A). [Leu^14^]exenatide-ABD decreased body weight gain by approximately 10%, comparable to the changes observed with exenatide infusion (Figure 3B). This reduction in weight gain was driven by comparable decreases in food intake (Fig. 3C) with [Leu^14^]exenatide-ABD and exenatide treatments, and in line with the reported anorexigenic action of exenatide [38].

### Pharmacokinetic Studies in Non-Human Primates

**Figure 4:**
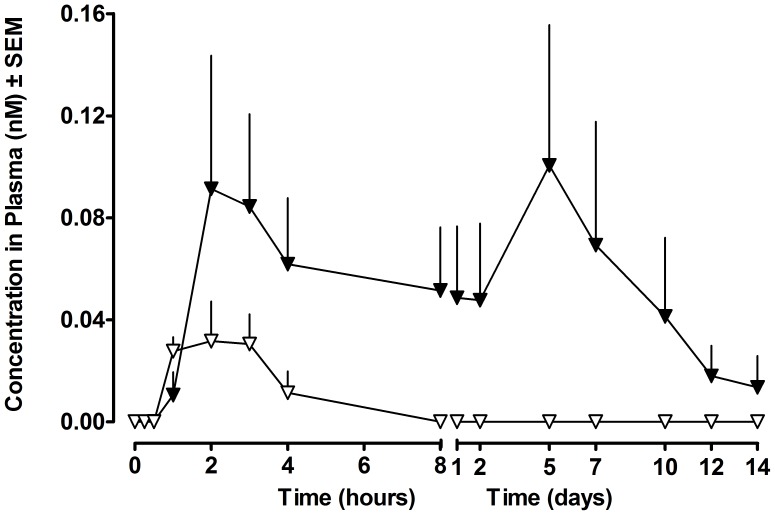
PK profile of orally dosed peptides in monkeys. [Leu^14^]exenatide-ABD (▾) and [Leu^14^]exenatide-ABP (▿), n = 3. Data are presented as mean ± SEM.

A single enteric coated capsule was given orally to each cynomolgus monkey. While absolute calculated bioavailability for both [Leu^14^]exenatide-ABP and [Leu^14^]exenatide-ABD was <1%, detectable plasma exposure of [Leu^14^]exenatide-ABP was <50 pM at all time points, whereas exposure of [Leu^14^]exenatide-ABD was >50 pM for 7 days and detectable for 14 days (Figure 4). This confirmed the extended circulation time of the ABD molecule, consistent with its association with monkey albumin (half life of 11–13 days), and the potential for oral uptake of this molecule when suitably formulated.

No adverse events were reported in the above animal studies.

## Discussion

The current study describes characterization of two novel long-acting exenatide analogs that were designed to have extended plasma circulation by virtue of their tight association with serum albumin, while retaining the key pharmacological actions of their parent hormone. [Leu^14^]exenatide-ABP is a fusion molecule that has [Leu^14^]exenatide conjugated to an albumin binding peptide *via* a Gly-Gly-Gly linker. [Leu^14^]exenatide-ABD is comprised of [Leu^14^]exenatide and an albumin binding domain from bacterial Streptococcus protein G that are linked *via* a Gly-Gly-Ser sequence.

Both molecules displayed extremely high affinity for serum albumin from various species. [Leu^14^]exenatide-ABD was found to bind to albumin with greater avidity than [Leu^14^]exenatide-ABP, and is comparable to un-conjugated ABD035 in its affinity. [Leu^14^]exenatide-ABD displayed a descending order of affinity for albumins from human, rat, monkey, dog and mouse, with a 50 fold difference in affinity for human (5 pM) and mouse (253 pM) albumins. Based on its picomolar affinities for albumins from various species and the presence of high concentration of serum albumin in blood, it is expected that [Leu^14^]exenatide-ABD will exist essentially in the bound state *in vivo*. Despite its tight binding to albumin, [Leu^14^]exenatide-ABD retained potent GLP-1 receptor functional activity in the presence of high concentrations of serum albumin (1%) of different species. This is surprising in part because liraglutide, a GLP-1 analog with much weaker albumin binding properties [29], [39], has been reported to display significant activity only when dissociated from albumin [40]. We speculate that the ABD moiety in [Leu^14^]exenatide-ABD also functions as a spacer to minimize undesirable steric interactions between exenatide and the bound albumin, thereby permitting the exenatide component to interact with the GLP-1 receptor. The calculated half-lives of [Leu^14^]exenatide-ABD and [Leu^14^]exenatide-ABP in rats was determined to be 16 hours and 11 hours respectively, while the half lives of the non-conjugated exenatide molecules were less than 30 minutes. The terminal half-lives of liraglutide ranging from 4–8 hrs has been reported in mice, rats, rabbits and monkeys (see European Medicines Agency Assessment Report for Victoza; Doc. Ref: EMEA/379172/2009) and is approximately 4 hrs in rats [41]. This is in contrast to the above-mentioned half-lives for [Leu^14^]exenatide-ABP and [Leu^14^]exenatide-ABD, which are in line with the stronger affinity of the peptides for albumin.

We then evaluated the pharmacokinetic profile of the engineered peptides in higher mammal species. Intravenous dosing of [Leu^14^]exenatide-ABP and [Leu^14^]exenatide-ABD in monkeys produced exposure of 2 days and greater than 14 days, respectively. In other studies, [Leu^14^]exenatide-ABD exposure after a single intravenous dose persisted up to four days in dogs (data not shown). The turnover of albumin in the cynomolgus monkey is about 11–13 days, and about 19 days in humans. Thus, allometric scaling combined with the observed strong affinity of [Leu^14^]exenatide-ABD for human albumin suggest that a duration of exposure of at least 14 days could be expected in human subjects. The shorter duration of exposure of [Leu^14^]exenatide-ABP in monkeys, coupled with its susceptibility to degradation in human plasma, led us to focus on [Leu^14^]exenatide-ABD for additional investigation in diabetic mice.

[Leu^14^]exenatide-ABP and [Leu^14^]exenatide-ABD were both full GLP-1 receptor agonists, albeit with somewhat reduced potency compared to native exenatide. A 100 fold loss of *in vitro* functional activity of [Leu^14^]exenatide-ABD compared to exenatide may be attributed to steric hindrance associated with the larger peptide chain. This finding is consistent with the reduced receptor potencies reported for liraglutide (replicated in our studies, Table 3) and albugon that are GLP-1 analogs derivatized with a fatty acid functionality or conjugated to human serum albumin, respectively. Despite the loss in *in vitro* potency, [Leu^14^]exenatide-ABD and [Leu^14^]exenatide-ABP retain the required exenatide-like activity to provide comparable glucoregulatory effects with longer duration of action in rodent models. It is known that while both native GLP-1 and exenatide show equivalent binding potency at the GLP-1 receptor [42], [43], exenatide exhibits significantly greater effects for glucose lowering than GLP-1 as a consequence of its greater metabolic stability [44]. This analogy can be extended to explain our findings with native exenatide and exenatide-ABD, where a lower *in vitro* potency of exenatide-ABD is more than compensated by its superior pharmacokinetic properties to afford comparable pharmacodynamic effects.

The extended duration of glucoregulatory benefits with these exenatide analogs was demonstrated in studies in normal and diabetic rodents. In an acute OGTT, [Leu^14^]exenatide-ABP, [Leu^14^]exenatide-ABD and the parent exenatide molecule significantly lower plasma glucose when administered just prior to the oral glucose challenge, with ED_50_ values of 3, 8 and 0.9 nmol/kg for [Leu^14^]exenatide-ABD, [Leu^14^]exenatide-ABP and [Leu^14^]exenatide, respectively. However, only [Leu^14^]exenatide-ABD and [Leu^14^]exenatide-ABP elicited significant reduction in plasma glucose when administered 24 h prior to the glucose challenge.

In additional studies with [Leu^14^]exenatide-ABD, robust HbA1c lowering was observed in diabetic *Lep^ob/ob^* mice, which received twice-weekly injections of [Leu^14^]exenatide-ABD for 4 week. These effects were significantly greater than those obtained with continuous infusion of exenatide at a total weekly dose matching the weekly dose of [Leu^14^]exenatide-ABD. The reduction of body weight gain seen with [Leu^14^]exenatide-ABD was comparable to that observed for continuous infusion of native exenatide.

Attempts for oral delivery of peptides has been limited to a few therapeutic molecules such as somatostatin [45], salmon calcitonin [46], insulin [47] and Sandimmune™, an approved immunosuppressant drug. The harsh acidic environment in the stomach and enzymatic hydrolysis in the gut conspire in the rapid degradation and loss of peptides. In addition, large molecular size and physicochemical properties adversely affect uptake, resulting in very low bioavailability. Despite these limitations, we carried out proof of concept pharmacokinetic study for oral delivery in non-human primates.

In the study, cynomolgus monkeys were dosed orally with a single enteric coated capsule containing [Leu^14^]exenatide-ABP or [Leu^14^]exenatide-ABD. Once the capsule transited the stomach, it was designed to dissolve at the neutral pH in the intestine to release its contents to facilitate the peptide’s permeation across the mucosal barrier and intestinal cell wall barrier. Surprisingly, relatively rapid uptake of [Leu^14^]exenatide-ABD in plasma was observed, and after about two hours, levels expected to be therapeutic based on exenatide exposure observed in the clinic (>50 pM) were achieved and were sustained over 7 days. It remains to be seen whether the flat pharmacokinetic profile and a relatively low C_max_ noted in our study will enhance patient tolerability in future studies. The absolute oral bioavailability of [Leu^14^]exenatide-ABD calculated in monkeys was <1%. Despite this relatively poor bioavailability, therapeutic concentrations of [Leu^14^]exenatide-ABD were attained in plasma, and its extended plasma residence support the potential for weekly oral dosing.

In summary, we have shown that fusion of an exenatide analog to albumin binding motifs of various origin provide novel long acting analogs [Leu^14^]exenatide-ABP and [Leu^14^]exenatide-ABD that display potent glycemic control and significantly prolonged duration of action compared to their parent hormone. These molecules bind albumin with high affinity while in circulation, leading to their extended duration of action. The analogs are significantly smaller in molecular size compared to long-acting GLP-1 clinical candidates that rely on Fc, albumin or XTEN conjugation for half life extension, are amenable to production by cost-effective recombinant processes, and hence offer the promise of superior manufacturing cost of peptide per therapeutic dose. In addition, their albumin binding affinities can be tuned for daily or even weekly delivery *via* different administration routes. Long exposure of [Leu^14^]exenatide-ABD obtained in non-human primates after oral delivery warrant further studies for optimization of the oral delivery formulations.
